# Leaf Induction Impacts Behavior and Performance of a Pollinating Herbivore

**DOI:** 10.3389/fpls.2021.791680

**Published:** 2021-12-17

**Authors:** Deidra J. Jacobsen, Robert A. Raguso

**Affiliations:** ^1^Department of Neurobiology and Behavior, Cornell University, Ithaca, NY, United States; ^2^School of Biological Sciences, University of Utah, Salt Lake City, UT, United States

**Keywords:** herbivore-induced plant volatiles, larval performance, *Manduca sexta*, *Nicotiana*, oviposition, plant resistance, pollinating herbivore

## Abstract

Flowering plants use volatiles to attract pollinators while deterring herbivores. Vegetative and floral traits may interact to affect insect behavior. Pollinator behavior is most likely influenced by leaf traits when larval stages interact with plants in different ways than adult stages, such as when larvae are leaf herbivores but adult moths visit flowers as pollinators. Here, we determine how leaf induction and corresponding volatile differences in induced plants influence behavior in adult moths and whether these preferences align with larval performance. We manipulated vegetative induction in four *Nicotiana* species. Using paired induced and control plants of the same species with standardized artificial flowers, we measured foraging and oviposition choices by their ecologically and economically important herbivore/pollinator, *Manduca sexta*. In parallel, we measured growth rates of *M. sexta* larvae fed leaves from control or induced plants to determine if this was consistent with female oviposition preference. Lastly, we used plant headspace collections and gas chromatography to quantify volatile compounds from both induced and control leaves to link changes in plant chemistry with moth behavior. In the absence of floral chemical cues, vegetative defensive status influenced adult moth foraging preference from artificial flowers in one species (*N. excelsior*), where females nectared from induced plants more often than control plants. Plant vegetative resistance consistently influenced oviposition choice such that moths deposited more eggs on control plants than on induced plants of all four species. This oviposition preference for control plants aligned with higher larval growth rates on control leaves compared with induced leaves. Control and induced plants of each species had similar leaf volatile profiles, but induced plants had higher emission levels. Leaves of *N. excelsior* produced the most volatile compounds, including some inducible compounds typically associated with floral scent. We demonstrate that vegetative plant defensive volatiles play a role in host plant selection and that insects assess information from leaves differently when choosing between nectaring and oviposition locations. These results underscore the complex interactions between plants, their pollinators, and herbivores.

## Introduction

Flowering plants face the crucial challenge of how to attract pollinators while deterring herbivores. This can be especially challenging when mutualists and antagonists are attracted to similar cues or signals that may indicate plant identity or resource quality. Understanding how plants strike this balance is vital to identifying the factors that facilitate plant-insect coevolution and constrain plant defensive and floral displays. Plants devote energy to many processes, including growth, herbivore avoidance, and reproduction. Because all these processes require resources, plant growth and defense are traditionally considered to be limited by energetic trade-offs (Herms and Mattson, 1992; Agrawal et al., 2010) and the different prioritization of resources can lead to trait variation across populations and species. Flowering plants rely on a variety of signals to attract pollinators as well as deter enemies. Floral volatiles have been shown to be a key component of flower attractiveness and pollinator choice (Raguso, 2008). For plants that rely partially or solely on outcrossing, reproduction often requires investment in large or dense floral displays to attract pollinators and ensure pollen transfer (Bell, 1985; Harder et al., 2004). Although pollinators are predicted to respond mainly to floral traits and herbivores are predicted to respond mainly to vegetative traits, vegetative and floral traits can have interactive effects on insect behavior (Späthe et al., 2013; Kárpáti et al., 2013).

Correlations between leaf and floral displays and defense-related metabolism impact how plants manage their interactions with herbivores and pollinators. Plant defenses are often controlled by the jasmonic acid pathway and secondary compounds in leaves and flowers can be produced constitutively or induced as a response to herbivore damage (Paré and Tumlinson, 1999). Additionally, herbivore damage reduces plant photosynthetic area and can decrease the overall pool of resources that plants have to devote to growth and reproduction. Because of this decrease and redirection of plant resources, herbivore damaged plants may suffer reduced pollinator visitation and reproductive/fitness consequences (Lehtilä and Strauss, 1999; Mothershead and Marquis, 2000). Herbivory has been shown to reduce plant attractiveness to pollinators by decreasing floral size or number (Kessler et al., 2011) or by increasing toxins present in the nectar or pollen (Adler et al., 2006; Kessler and Halitschke, 2009; Stevenson et al., 2016). Herbivory may also alter floral scent emission in ways that affect pollinator attraction and behavior, but these effects are varied across studies and species, ranging from decreased floral scent (Kessler et al., 2010; *Nicotiana attenuata*) to increased floral scent (Theis et al., 2009), or unchanged floral scent (Effmert et al., 2008; *Nicotiana suaveolens*; Reisenman et al., 2013; *Datura wrightii*) following insect damage. Temporal and diel variation in emission rate/timing of leaf and floral plant volatiles may reflect the different diel activity patterns of mutualistic and antagonistic insects and minimize the costs of deterring herbivores on pollinator attraction (Euler and Baldwin, 1996; De Moraes et al., 2001; Reisenman et al., 2013; Zhou et al., 2017).

Although there are clear interactions between herbivore leaf damage and floral traits important to pollinators, few studies have examined the direct impact of leaf damage on pollinator behavior and whether pollinators use leaf volatiles for feeding and oviposition cues. The importance of leaf and floral chemicals on an insect’s behavior is likely dependent on the extent to which the insect encounters either type of tissue, and this may vary throughout an insect’s development. In pollinating-herbivore systems, adults act as pollinators but larvae of the same insect species are herbivores, acting antagonistically against the plant species the adults pollinate (Adler and Bronstein, 2004; Irwin, 2010). Pollinating-herbivore systems are common across lepidopteran species (reviewed by Altermatt and Pearse, 2011) and similar relationships are seen in other floral visitors, such as sawflies and thrips (Wäckers et al., 2007) and we should expect these species to assess leaf and floral cues because of their interactions with both types of plant tissues.

While floral volatiles are known to influence adult preference and attraction in pollinating herbivores, these insects may pay additional attention to cues of leaf quality or defense for information that floral volatiles may not provide, such as whether that plant is a high-quality site for oviposition/offspring. Whether offspring deposited on undamaged plants have increased or reduced fitness depends on a variety of factors, including natural enemy prevalence and toxicity of the secondary compounds (Ohsaki and Sato, 1994; López-Ortega and Williams, 2018). Female tobacco hornworm moths (*Manduca sexta*) lay more eggs on host plants with higher emissions of floral volatiles (Kessler et al., 2015). This relationship between floral volatiles and oviposition preference may reflect plant quality if plants that produce high levels of floral volatiles also produce high quality leaf tissue for herbivores. Conversely, the pattern may not reflect plant quality but may instead reflect the strength of the signal if floral volatiles tend to be stronger and travel farther than leaf volatiles. In a wind tunnel experiment manipulating floral odor and vegetative olfactory background, the flight responses of *M. sexta* moths were stronger in response to the combined odors of host plant leaves and flowers but only when the leaves and flowers came from the same species (Kárpáti et al., 2013), suggesting that vegetative as well as floral odor is an important determinant of insect feeding and oviposition choices.

Using a common pollinating-herbivore, *Manduca sexta* and its Solanaceous host plants, we tested the predictions that vegetative defenses impact adult behaviors and that adult foraging and oviposition decisions are correlated. Some, but not all, tobacco species show a positive correlation between floral and vegetative defenses (Adler et al., 2012); therefore, we predicted that female *M. sexta* would not rely solely on floral cues to predict leaf defenses and that vegetative cues would inform adult foraging and oviposition choices. In each of four *Nicotiana* species, we examined constitutive and induced anti-herbivore resistance, the role of vegetative resistance on adult foraging and oviposition choice, and volatile differences among control and induced plants of each species. We show that larval growth was higher on uninduced plants, which had lower levels of anti-herbivore resistance and lower volatile emissions. Adult oviposition choices aligned with offspring performance, such that more eggs were laid on uninduced plants. However, foraging decisions were uncorrelated with oviposition preference and larval performance. *Manduca sexta* adult females either exhibited no foraging preference for control versus induced plants or preferred to forage from artificial flowers on an induced vegetative background. These results provide important evidence that pollinating herbivores use not only floral traits but also plant vegetative defensive volatiles/cues to inform foraging and oviposition decisions.

## Materials and Methods

### Plant Culture and Induction Treatments

**FIGURE 1:**
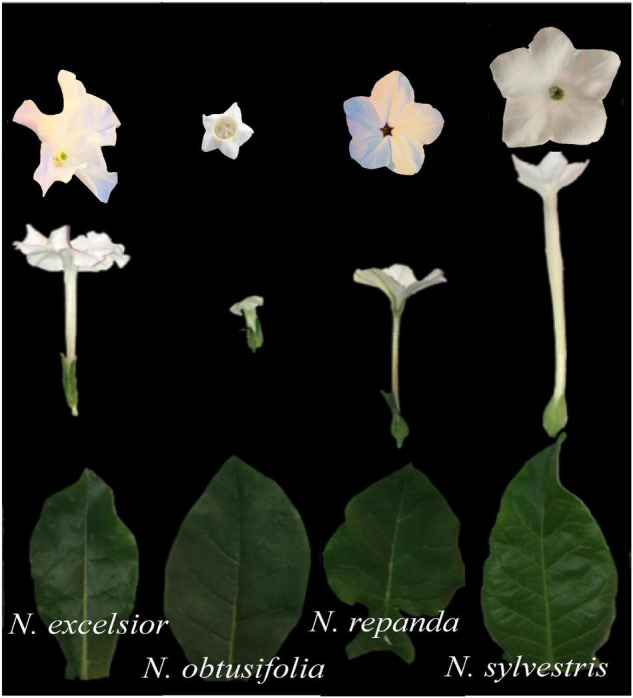
Flower and leaf morphology of *Nicotiana excelsior, N. obtusifolia, N. repanda*, and *N. sylvestris.*

To evaluate how vegetative plant defenses affect insect adult preference and offspring performance, we selected four *Nicotiana* species that are predicted or already known to vary in their defensive chemistry and resistance to the pollinating herbivore *Manduca sexta* (Baldwin and Ohnmeiss, 1993; Laue et al., 2000; Voelckel et al., 2001). Across *Nicotiana*, leaf and floral defenses are decoupled in many species and therefore pollinating herbivores may assess both floral and leaf defenses to choose feeding and oviposition locations (Adler et al., 2006; Sharp et al., 2009; Adler et al., 2012). The four species/accessions used for this study were grown from seeds obtained from the Germplasm Resources Information Network (GRIN): *N. excelsior* (Australia, 224063 TW 46), *N. obtusifolia* (= *N. trigonophylla* in previous literature; MD, United States, 555543 TW 98), *N. repanda* (MD, United States, accession 555552 TW 110), and *N. sylvestris* (MD, United States, accession 555570 TW 137) (Figure 1). Plants for all trials and treatments were grown in the same manner and separate plants were used for the four different assays (larval growth, adult foraging, adult oviposition, and plant volatile collection).

Plants of each species were grown in a greenhouse on a 15:9 light:dark cycle at 24 ± 2°C during light hours and 22 ± 2°C during dark hours. Seeds were germinated in individual soil cells (Lambert LM-111 all-purpose soil mix, Lambert Peat Moss, Québec, QC, Canada) and were fertilized five days a week with a 21-5-20 fertilizer (nitrogen-phosphate-potash) (Jack’s professional LX water soluble fertilizer, J.R. Peters Inc., Allentown, PA, United States). Seedlings were transplanted to 20 cm diameter pots and were grown for 3–6 weeks until they reached the large rosette/pre-flowering bolting stage, at which point they were used for the respective assays.

Twenty-four hours before each assay, size-matched plants were assigned to either control treatments (constitutively defended) or induction treatments. Exogenous jasmonates are commonly used to experimentally mimic a signal of herbivore damage and have been shown to be effective in *Nicotiana* species (Baldwin, 1996). Therefore, we used jasmonic acid to induce anti-herbivore resistance in a controlled manner across species without causing physical damage or tissue removal (which would serve as a visual cue of damage). Plants in the induction treatment were sprayed with 10 mL of 1 mMol JA in deionized water and control plants were sprayed with an equivalent amount of an ethanol vehicle dissolved in deionized water. After treatment, control and induced plants were separated in the greenhouse to avoid volatile communication and induction of control plants.

### *Manduca sexta* Rearing for Larval Performance and Adult Preference Assays

*Manduca sexta* larvae and adults came from a colony at Cornell University (Ithaca, NY, United States) that was originally established from laboratory-reared and wild moths collected in AZ, United States (Stillwell and Davidowitz, 2010). Larvae were reared communally on a cornmeal-based artificial diet (Broadhead and Raguso, 2021) in an environmental chamber at 24°C and 40–60% relative humidity on a 16:8 light:dark cycle. At the final larval instar, the pre-pupae were transferred to wooden boxes to pupate (Yamamoto, 1969). Following sclerotization, pupae were removed from the boxes and stored under the same conditions as larvae until wing patterning was visible through the pupal case (2–3 days prior to adult eclosion), at which time pupae were moved to the respective conditions described below for the foraging and oviposition assays.

### Larval Growth Assays for Measurements of Plant Anti-herbivore Resistance

For each plant species, we measured biologically relevant anti-herbivore resistance using *Manduca sexta* larval growth assays as a proxy for the level of constitutive and induced resistance in the leaves. Because adult oviposition choice affects neonate and young larval feeding site, these leaf feeding trials were done using early second instar larvae. To increase hunger, larvae were removed from artificial diet 1 h before being placed on the leaf.

Two leaves from each control (constitutive) and induced plant were placed upside down in individual lidded 2 oz containers. A piece of wet filter paper under each leaf prevented leaf desiccation during the trials. Larvae were weighed immediately prior to being placed on the leaf and after 24 h of feeding. The relative weight change was calculated for each larva as: log (final weight/initial weight) and was averaged over two larvae per plant (*N* = 5 plants per species and treatment). Larvae that died or molted during the trial were removed from the analysis because molting causes a temporary cessation of feeding unrelated to the experiment.

For these measurements, herbivore growth and plant resistance are inversely related. In contrast to plant defense–which measures the fitness benefit from the plant’s perspective–plant resistance is measured from the herbivore perspective (Karban and Myers, 1989). Therefore, a high herbivore growth rate indicates a low level of plant resistance and vice versa. Plant constitutive and induced resistance values were calculated as: 1-(average relative moth growth rate) for each treatment (Haak et al., 2014). Plant constitutive resistance was subtracted from induced resistance levels to calculate plant inducibility levels (Morris et al., 2006).

### Adult Female Foraging Choice Assays

To test for a role of leaf resistance on adult foraging preferences, we tested whether adult female moths preferred to nectar from artificial flowers on a control (constitutively defended) vegetative background or an induced vegetative background. *Manduca sexta* adults feed by hovering above flowers and extending their proboscis into the nectar. This feeding behavior is often preceded by probing of the flowers and/or leaves of the plant and foraging choice is based on a variety of visual and olfactory information (Goyret et al., 2007). Female adult *M. sexta* interact with flowers (for foraging) and leaves (for oviposition) but male adult moths only visit flowers. Therefore, we used only females for these assays as they may respond more strongly to vegetative volatiles than males (Sharp et al., 2009).

Two to three days prior to eclosion, female pupae were transferred to holding cages in a room under a reversed photoperiod (16:8 light dark cycle, with lights off from 8 am-4 pm) at 23 ± 4 °C and relative humidity > 20 percent. Moths were unfed and unmated to encourage feeding rather than oviposition behaviors. On the third day following emergence, moths were assayed during the first three hours of the dark phase.

For each conspecific pair of induced versus control plants, an individual female moth was placed on the floor of a large enclosure (1.7 × 1.7 × 1.7 m), equidistant and between the two plants (one control, one induced). An artificial, 5-lobed conical white paper flower (designed to resemble an average tobacco flower) was staked in each pot above the vegetative plant. A 1.5 mL tube with 1.25 mL of 20 percent sucrose solution was sunk down flush with the paper at the center of each flower. This volume of sugar solution was high enough that most moths were satiated and stopped foraging after one flower. Two nightlights (< 0.01 μmol of photons m^–2^s^–^^1^) suspended over the enclosure illuminated each plant from above.

The behaviors scored during each trial were: time to take flight (warmup), time spent flying before foraging (latency), and time spent feeding at each flower (foraging time). A foraging decision was scored when a moth extended its proboscis into the paper flower and hovered over the plant for > 5 s. Foraging was confirmed by measuring the amount of sucrose consumed. If moths did not fly or choose a flower within 20 min, they were removed from the arena. Trials continued until *N* = 30 moths per plant species had chosen to forage from either the control or induced plant.

### Adult Female Oviposition Assays

To test for a role of vegetative defensive status on adult oviposition preferences, we counted the number of eggs a female moth laid on a control (constitutively defended) vegetative plant and an induced vegetative plant in a 24-h binary choice assay. Two to three days prior to eclosion, female and male pupae were transferred to sex-specific cages (45 × 45 × 45 cm; Bioquip Inc., Rancho Dominquez, CA, United States) in a greenhouse with a 15:9 light:dark cycle set to 27°C during light hours and 21°C during dark hours. On the third day post-eclosion, females and males were moved to a mating cage with a ratio of two males per female. Each cage contained a cup providing 20 percent sucrose solution *ad libitum*. After 24 h in the mating cage (on the morning of the fourth day), individual female moths were moved to larger cages (61 × 61 × 91.5 cm; Bioquip Inc., Rancho Dominquez, CA, United States) that contained a vegetative induced plant and a control plant on opposite sides. After 24 h, the female was removed from the cage and the number of eggs deposited on each plant were counted and allowed to hatch to verify the female had mated. Females that died during the assay or had not mated were discarded and trials continued until *N* = 35 moths per plant species had laid fertilized eggs during the oviposition choice test.

### Volatile Collection

We used volatile collections and gas chromatography to identify chemical differences among the leaves and flowers of control and induced plants of each species. Leaf volatile emissions were collected from control and induced plants of each of the four species under both day and night conditions. This was done using a volatile collection system from Analytical Research Systems, Inc. (Gainesville, FL, United States) using collection methods modified from Heath and Manukian (1994).

Volatile collections were done in a greenhouse under 16:8 light:dark conditions with supplemental light provided to plants during the day to maintain photosynthetically active radiation (PAR)-values > 150 μmol photons m^–2^ s^–1^. To avoid volatile changes due to temperature, isothermal conditions were maintained (24.5°C) during both day and night. Two plants of the same species and size (one control and one induced 24 h prior) were run simultaneously in separate Pyrex^®^ dome collection chambers (25 cm diameter × 55 cm tall). An aluminum guillotine base closed around the stem of the plant separated the leaves in the collection chamber from the soil below. Air first passed through a charcoal air filtration system with Teflon tubing before being pumped into each plant’s collection chamber at a rate of 5 L/min (1.75 L/min wet + 3.25 L/min dry air flow).

Two five-hour collections were obtained from each plant: a day collection (9 am–2 pm) and a night collection (9 pm–2 am). Thus, day and night measurements came from the same individual and we were able to compare control versus induced emissions during both day and night conditions. To maintain airflow and prevent buildup of volatiles or condensation in the collection chambers between the day and night collections, a run was programmed from 3 pm–8 pm but these samples were not analyzed. Additional control (“blank”) collections were run using soil pots under the stage that did not contain plants. Because foraging assays were done in the dark during daytime hours on a reversed moth photoperiod, additional volatile collections were done during daytime hours from plants in the dark. Volatile emissions in tobacco plants can vary based on diel rhythms (day and night; De Moraes et al., 2001; Raguso et al., 2003) or photoperiod (dark and light; He et al., 2021). When volatile emissions differed based on time of day (morning or night) for a particular species, this additional volatile collection allowed us to determine whether the “dark day” condition more closely mimicked morning or evening volatiles.

During each sampling period, air was pulled from the collection chamber through Tygon tubing into the glass collection traps containing 25 mg of Alltech Super-Q^®^ using a vacuum pull rate of 0.5 L/min. These volatile collections were automated using the timed-event sequencing software (programmable logic controller, Analytical Research Systems, Inc.). After collection, the traps were wrapped in nylon resin oven bagging (Reynolds Kitchens™) and aluminum foil and frozen at −20°C for storage. Traps were eluted with 200 μL GC-MS purity hexane and the sample was evaporated down to 50 μL using a stream of nitrogen, from which 1 μL aliquots were injected and analyzed separately on two gas chromatograph instruments (GC; Shimadzu Scientific Instruments, Inc.), one with a mass spectrometer (GC17A with a QP5000 MS, Shimadzu) as a detector, and the other (GC2014) with a flame ionizing detector (FID, Shimadzu). MS was used primarily to identify the different volatile organic compounds (VOCs), using electrical ionization (EI; 70 eV), single quadrupole mass spectra, whereas the FID was used to quantify relative emission rates of each identified VOC, using 5 μL of 0.05% tridecane as an internal standard. Polar capillary GC columns (ethylene glycol stationary phase; 30 m length, 0.25 mm internal diameter, 0.25 μm film thickness) were used to separate trapped volatiles on each instrument (Stabilwax, Restek, Inc., for GC-MS; Econo-Cap ECwax, Grace, Inc., for GC-FID).

### Volatile Identification and Analysis

Gas chromatography–mass spectrometry (GC-MS) chromatogram peaks were used for compound identification and GC-FID peaks were used to quantify peak abundance. A blend of n-alkanes (C7-C30) was injected under the same chromatographic conditions as the volatile samples, and retention times were used to calculate Kovats Retention Indices (KI) for all volatile peaks, as described by Schlumpberger and Raguso (2008). Retention time for each GC-MS peak was noted at the start of the inflection point (Jennings et al., 1997). When authentic standards were not available for compound identification, an identification was based on a match to published KI-values on an equivalent column (NIST webbook^1^) and the best suggested match with over 80% confidence in the MS library.

Gas chromatography- flame ionizing detector (GC-FID) peak detection and auto-integration were done using the default parameters, with the maximum linear shift set to 0.05, the maximum distance between the peak and mean across samples set to 0.03, and the minimum expected distance between peaks set to 0.03. ASCII files containing the data parameters were exported for peak alignment across samples (LabSolutions software). GCalignR in R v3.3.1 statistical software was used to align peaks across samples and species (Ottensmann et al., 2018; R Core Team, 2013). Because there is a small amount of drift as more samples are run on the same column (∼0.25 s difference in retention time across samples), multiple alignments were run with different combinations of samples to verify the alignments were robust to sample order on the FID. The retention time of the internal standard and the alkane ladders on the respective machines were used to aid in peak matching between the GC-MS and GC-FID. After peak alignment across samples, peaks were further cleaned up and matched to the GC-MS spectra manually in Microsoft Excel. Auto-identified FID peaks in the following categories were excluded: peak area less than 2x higher than the control collections, peaks present in only one sample, peaks that were indistinguishable from the baseline, and peaks without a corresponding peak match on GC-MS (based on KI and visual inspection of the chromatograms). For each sample, peak areas from the GC-FID were normalized by dividing by the peak area of the internal standard. These normalized peak areas were used for multivariate statistical comparisons among species and treatments. Normalized peak areas for all compounds present in a sample were summed to calculate the total volatile production. The emission rate for each plant was calculated as: total volatile production x amount of internal standard injected (34 ng) × sample volume (55 μL) / timespan of volatile collection (5 h).

## Statistical Analysis

All analyses were done using R v. 3.3.1 (R Core Team, 2013) and each species was analyzed separately for the larval growth and adult behavioral data. One-tailed two sample *t*-tests were used to test whether larval growth was lower on plants induced with jasmonic acid than on plants with constitutive levels of resistance (t.test() in the stats package). Prior to analysis, normality was confirmed using a Shapiro-Wilk test (shapiro.test() in the stats package) and homogeneity of variance was confirmed with a Levene test (leveneTest() in the car package (Fox and Weisberg, 2019).

To compare choice for either control or induced plants in foraging assays, chi-squared tests (chisq.test() in the stats package) were used to determine if the adult females fed from the artificial flower on either the conspecific control or induced plant more than expected (i.e., significantly more than the 50/50 choice expected by change in the binary choice assay). Chi-squared analyses were also used to test whether the plant species in the conspecific binary choice trials affected a moth’s likelihood to forage from either flower. General linear models with type three sums-of-squares were used to test whether plant species or nectar choice (or the interaction between them) affected the length of time a moth spent in three foraging activities: warm-up time before taking flight, flight time before foraging, or time spent at the artificial flower chosen.

To determine if mated adult females showed an oviposition preference for conspecific control or induced plants, binomial generalized linear models (glm() in the stats package) were used to test whether the numbers of fertilized eggs laid on control and induced plants in the binary assays were significantly different from each other.

To visualize variation among species and treatments in plant leaf chemistry, we used non-metric multidimensional scaling (NMDS) with Bray-Curtis matrices (metaMDS() in the vegan package) (Oksanen et al., 2017). Centroid values were calculated for the two-dimensional plots (NMDS1 and NMDS2) (envfit() in vegan). Ellipses were generated using covariance matrices (veganCovEllipse() and cov.wt() in vegan) and plotted using the ggplot2 package (Wickham, 2009). Total plant volatile emission rates were compared across species and treatments using ANOVA and Tukey *post hoc* comparisons (aov() in car) (Fox and Weisberg, 2019).

To identify which volatile compounds were responsible for the significant groupings identified in the NMDS analyses, we used indicative compound analyses. Indicative compound analyses are an application of indicator species analyses (Dufrene and Legendre, 1997; Bakker, 2008) for chemical data, as described in Heuskin et al. (2014). These analyses were run using the multipatt() command in the indicspecies package (De Cáceres and Legendre, 2009) with association function “IndVal.g.” Groupings were restricted for the induction treatment to identify compounds that are associated with either control or induced treatments. Compounds were allowed to group with multiple conditions for the species and time/light analyses to allow identification of compounds that are present in multiple species or throughout different diurnal/light rhythms. The association indices yield two values between 0 and 1, the first (A) indicates the probability that a specific compound is present in the species or treatment in question (i.e., *A* = 1 means that the volatile compound is found only in that species or treatment). The second value (B) indicates the probability that compound is found in a specific species or treatment (i.e., *B* = 1 means that a specific compound is present in all the samples of a particular species or treatment) (Lemieux-Labonté et al., 2017). *P*-values were corrected for multiple testing (p.adjust() in the stats package) and both raw and false discovery rate (FDR) adjusted *P*-values are presented in the statistical tables.

## Results

### Larval Growth Assays Show Variation in Plant Anti-herbivore Resistance Across Species

**FIGURE 2:**
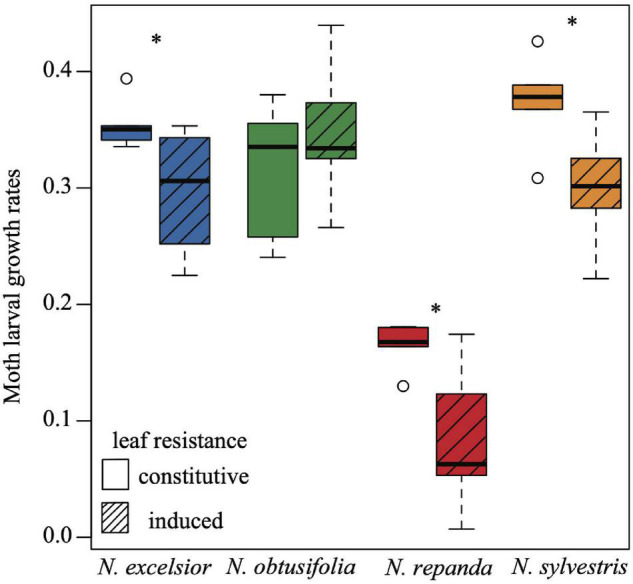
Larval growth rates of *Manduca sexta* differ across *Nicotiana* species and among plants with constitutive or induced levels of resistance. Moth larval growth rates were calculated as log (final growth rate/initial growth rate) over a 24-h cut leaf feeding assay on control (constitutive) and induced plants of each of the four species. Larval growth rates were higher on control plants for *N. excelsior, N. repanda*, and *N. sylvestris* but not *N. obtusifolia* (one-tailed two-sample *t*-tests *N. excelsior P* = 0.030; *N. obtusifolia P* = 0.790; *N. repanda P* = 0.015; *N. sylvestris P* = 0.020). Asterisks indicate a significant difference between control and induced treatments within a species (*P* < 0.05).

The four *Nicotiana* species varied in their overall resistance levels to *Manduca sexta* larvae. *Nicotiana repanda* had the highest overall resistance (Figure 2). Three of the species showed significantly inducible plant resistance (*N. excelsior*, *N. repanda*, and *N. sylvestris* but not *N. obtusifolia*). This was evidenced by lower *M. sexta* larval growth during the 24-h feeding assays on leaves from plants induced with jasmonic acid compared with leaves from constitutively defended plants (one-tailed two-sample *t*-tests: *N. excelsior t*_8_ = −2.174, *P* = 0.030; *N. obtusifolia t*_8_ = 0.851, *P* = 0.790; *N. repanda t*_8_ = −2.624, *P* = 0.015; *N. sylvestris t*_8_ = −2.442, *P* = 0.020).

### Plant Vegetative Induced Resistance Affects Adult *Manduca sexta* Foraging Preference

**FIGURE 3:**
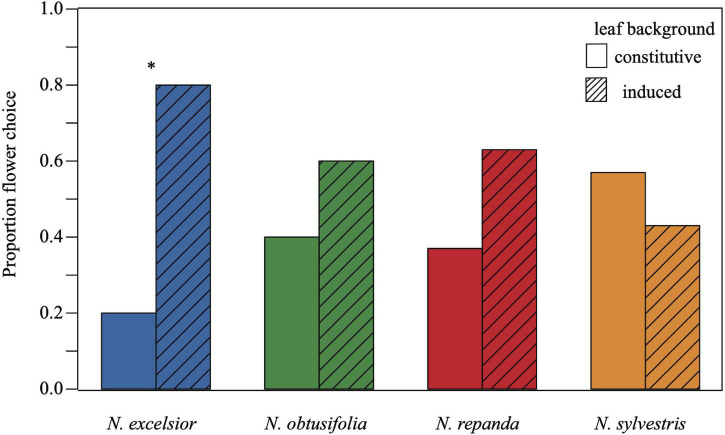
A greater proportion of virgin *Manduca sexta* females chose to forage from the artificial flower on the induced *Nicotiana excelsior* vegetative background compared to the control (constitutive) vegetative background. Each moth was tested only once, and choice represents the first flower chosen for nectaring during the conspecific binary choice assays. Twenty-four out of thirty females chose to forage from the artificial flower on the induced *N. excelsior* plant while only six chose the artificial flower on the uninduced plant (chi-squared *P* = 0.001). Moths did not exhibit a foraging difference in the other three *Nicotiana* species (*N* = 30 trials, chi-squared *P* > 0.01 for *N. obtusifolia N. repanda*, and *N. sylvestris*). Asterisks indicate a significant difference between control and induced treatments within a species (*P* < 0.05).

In one of the four plant species (*N. excelsior)*, virgin adult female *M. sexta* chose to forage first from the artificial flower on the induced vegetative background compared with the identical flower on a conspecific control background (Figure 3). Across all trials, over 76 percent of the moths flown made a foraging decision (*N* = 33–44 total trials per species were run to achieve *N* = 30 successful foraging choices) and the percent of successful moth foraging trials was not significantly different across the four host plant species ($\chi_{3}^{2}=7.17$, *P* = 0.067).

There was no difference in moth pre-foraging flight behaviors across the trials with different host plant species or between moths that made different foraging choices. Time to begin flying (warm-up time) did not differ based on the plant species or whether the moth ultimately chose to nectar from the artificial flower on the uninduced or induced vegetative background (GLM: species $\chi_{3}^{2}=1.11$, *P* = 0.774; nectar choice $\chi_{1}^{2}=0.570$, *P* = 0.451; species*choice $\chi_{3}^{2}=2.062$, *P* = 0.560). Likewise, time spent flying before choosing a foraging location did not differ based on species or foraging choice (GLM: species $\chi_{3}^{2}=1.22$, *P* = 0.747; nectar choice $\chi_{1}^{2}=0.74$, *P* = 0.390; species*choice $\chi_{3}^{2}=3.38$, *P* = 0.337). Additionally, the time spent at the chosen artificial flower was similar for all the species and foraging choices (GLM: species $\chi_{3}^{2}=4.73$, *P* = 0.192; nectar choice $\chi_{1}^{2}<0.001$, *P* = 0.984; species*choice $\chi_{3}^{2}=0.619$, *P* = 0.892).

Although pre-foraging behaviors and time spent foraging were similar regardless of the plant species or vegetative induction status, we found that moths preferentially foraged from the artificial flower on the induced vegetative background over the uninduced background in trials with one of the four plant species. For foraging trials on an *N. excelsior* vegetative background, 80 percent of the *M. sexta* females nectared first from the artificial flower on the induced plant ($\chi_{1}^{2}=10.8$, *P* = 0.001). For the other three species, there was no difference in the percent of moths choosing the control versus the induced plant (*N. obtusifolia*: $\chi_{1}^{2}=1.2$, *P* = 0.273; *N. repanda*: $\chi_{1}^{2}=2.13$, *P* = 0.144; *N. sylvestris*: $\chi_{1}^{2}=0.53$, *P* = 0.465).

### Adult Female *Manduca sexta* Oviposit More Eggs on Leaves of Uninduced Plants

**FIGURE 4:**
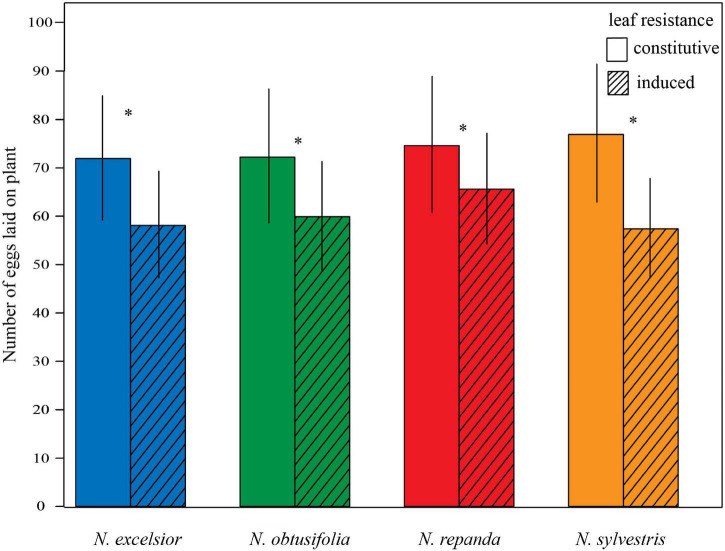
*Manduca sexta* preferentially oviposited on uninduced plants. In 24-h binary choice oviposition assays, *Manduca sexta* laid more eggs on a control (constitutive) vegetative plant compared to a conspecific induced plant. Four *Nicotiana* species were tested in separate uninduced versus induced oviposition assays (binomial GLM *P* < 0.001 for each species). Bars represent mean ± 2 standard errors. Asterisks indicate a significant difference between control and induced treatments within a species (*P* < 0.05).

During the 24-h oviposition assays with mated females, more eggs were laid on control plants than on induced plants (Figure 4). Overall, 84 percent of the moths laid fertilized eggs and 38–44 total trials per species were run to reach *N* = 35 successful female oviposition trials. For all *Nicotiana* species, egg counts were significantly higher on the control plant than on the induced plant (GLM: *N. excelsior z*_34_ = 7.061, *P* < 0.001; *N. obtusifolia z*_34_ = 6.313, *P* < 0.001; *N. repand*a *z*_34_ = 4.553, *P* < 0.001; *N. sylvestris z*_34_ = 9.884, *P* < 0.001).

### Volatile Chemistry Differs Across the Four *Nicotiana* Species

**FIGURE 5:**
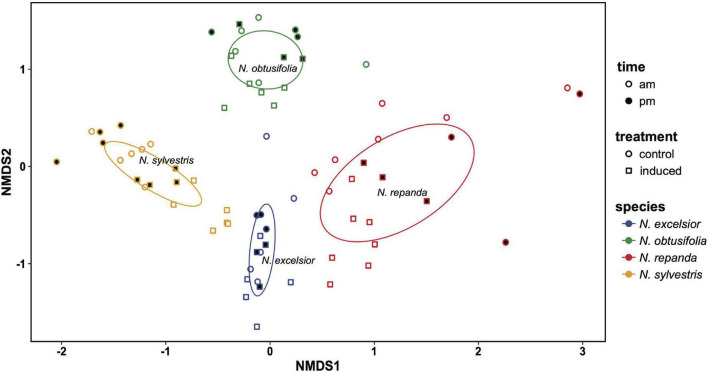
Non-linear multidimensional scaling (NMDS) plot showing clustering associated with species differences in leaf volatile chemistry in the four *Nicotiana* species. Ellipses were generated using covariance matrices and are color coded by species. Shape of each point indicates whether leaves were induced with jasmonic acid (square) or control (constitutive; circle). Point fill indicates the time of day volatile emission was measured (morning = unfilled points and evening = filled/black points). 2D stress = 0.197, species *r*^2^ = 0.745, *P* = 0.001.

**FIGURE 6:**
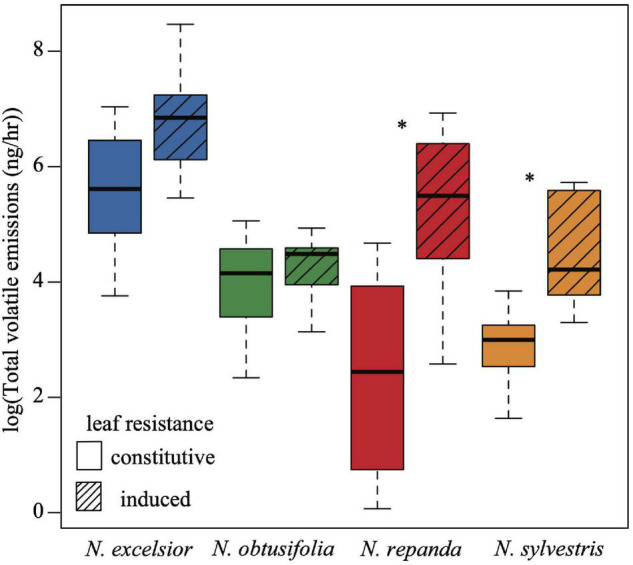
Overall volatile emission rates (ng total scent / h) varies among species and with induction. Total scent emission rate was higher in *N. excelsior* than in *N. repanda*, *N. obtusifolia*, and *N. sylvestris* (ANOVA *P* < 0.001). Induced plants of *N. repanda* and *N. sylvestris* had higher overall emission rates than control (constitutive) plants (ANOVA and *post hoc* Tukey test: *N. repanda P*_adjusted_ < 0.001 and *N. sylvestris P*_adjusted_ = 0.02). Asterisks indicate a significant difference between control and induced treatments within a species (*P* < 0.05).

We detected fifty leaf volatile compounds across the four *Nicotiana* species (Supplementary Table 1). Although there was overlap in the species volatile profiles, the NMDS clusters show species-specific differences in volatile composition (Figure 5). *Nicotiana excelsior* had the most complex blend of volatiles (Table 1) and the highest total volatile emission rate (Figure 6). These compounds largely consisted of common green leaf volatiles (GLVs), including (*Z*)-3-hexen-1-ol and four associated esters, along with monoterpenoids (β-myrcene, linalool) and sesquiterpenoids (β-caryophyllene, caryophyllene oxide, (*E,E*)-α-farnesene, farnesol isomers) (Supplementary Table 1). We also identified small amounts of aromatic (e.g., methyl benzoate, 2-phenylethanol) and nitrogenous VOCs (e.g., benzyl cyanide, 2-methylbutyl aldoxime) typical of flowers, along with methyl esters of long-chain fatty acids (Supplementary Table 1). Finally, we found two diterpene-related compounds typical of wounded tobacco foliage, including (*E,E*)-4,8,12-trimethyltrideca-1,3,7,11-tetraene (TMTT) and 7,11,15-trimethyl-3-methylidenehexadec-1-ene (neophytadiene; Supplementary Table 1).

**TABLE 1:** Volatile compounds differed among *Nicotiana excelsior* (Nex), *N. obtusifolia* (Nob), *N. repanda* (Nre), and *N. sylvestris* (Nsy).

COMPOUND	CLASS	SPECIES/GROUP	A, B, STAT	*P*	*FDR P*
(*Z*)-3-HEXEN-1-OL	GLV	*Nex, – – –, – – –, – – –*	0.84, 1.00, 0.91	< 0.001*	<0.001*
2-METHYLBUTYL-ALDOXIME	Nitrogenous	*Nex, – – –, – – –, – – –*	0.83, 0.63, 0.72	< 0.001*	<0.001*
(*Z*)-3-HEXENYL BENZOATE	Aromatic	*Nex, – – –, – – –, – – –*	0.91, 0.94, 0.93	< 0.001*	<0.001*
UNKNOWN METHYL ESTER 2		*Nex, – – –, – – –, – – –*	0.78, 0.75, 0.77	< 0.001*	<0.001*
BENZOIC ACID ESTER	Aromatic	*Nex, – – –, – – –, – – –*	0.69, 0.75, 0.72	0.001*	0.002*
(*Z*)-3-HEXENYL BUTYRATE	GLV	*Nex, – – –, – – –, – – –*	0.98, 1.00, 0.99	< 0.001*	<0.001*
M/Z 43(100), 67(75), 41(54), 82(46), 71(34), 55(15), 42(13), 57(9), 40(7), 68(6)	GLV	*Nex, – – –, – – –, – – –*	0.98, 0.69, 0.82	< 0.001*	<0.001*
(*Z*)-3-HEXENYL ISOVALERATE	GLV	*Nex, – – –, – – –, – – –*	0.84, 1.00, 0.92	< 0.001*	<0.001*
BENZYL ISOCYANIDE	Nitrogenous	*Nex, – – –, – – –, – – –*	0.97, 0.94, 0.95	< 0.001*	<0.001*
(*Z*)-JASMONE	GLV	*Nex, – – –, – – –, – – –*	0.64, 0.69, 0.67	0.020*	0.023*
BENZENE NITRILE	Nitrogenous	*Nex, – – –, – – –, – – –*	1.00, 0.81, 0.90	< 0.001*	<0.001*
(*Z*)-β-OCIMENE	Monoterpene	*Nex, – – –, – – –, – – –*	1.00, 0.25, 0.50	0.002*	0.002*
(*E*)-β-OCIMENE	Monoterpene	*Nex, – – –, – – –, – – –*	0.91, 1.00, 0.95	< 0.001*	<0.001*
LINALOOL	Monoterpene	*Nex, – – –, – – –, – – –*	0.74, 0.75, 0.75	0.002*	0.003*
(*E*)-FURAN LINALOOL OXIDE	Monoterpene	*Nex, – – –, – – –, – – –*	1.00, 0.56, 0.75	< 0.001*	<0.001*
(*E*)-α-BERGAMOTENE	Sesquiterpene	*Nex, – – –, – – –, – – –*	0.82, 1.00, 0.91	< 0.001*	<0.001*
α-MUUROLENE	Sesquiterpene	*Nex, – – –, – – –, – – –*	0.87, 0.63, 0.74	< 0.001*	<0.001*
(*E,E*)-α-FARNESENE	Sesquiterpene	*Nex, – – –, – – –, – – –*	0.84, 0.94, 0.89	< 0.001*	<0.001*
OCIMENE EPOXIDE ISOMER		*Nex, – – –, – – –, – – –*	0.95, 0.63, 0.77	< 0.001*	<0.001*
M/Z 69(100), 41(92), 91(62), 93(45), 77(36), 42(32), 53(32), 92(27), 79(23), 67(22)		*Nex, – – –, – – –, – – –*	0.96, 0.44, 0.65	< 0.001*	<0.001*
FARNESOL ISOMER 1	Sesquiterpene	*Nex, – – –, – – –, – – –*	0.91, 0.81, 0.86	< 0.001*	<0.001*
FARNESOL ISOMER 2	Sesquiterpene	*Nex, – – –, – – –, – – –*	0.74, 0.88, 0.81	< 0.001*	<0.001*
UNKNOWN SIMILAR TO METHYL LINOLEATE	Aliphatic	*Nex, – – –, – – –, – – –*	0.99, 0.88, 0.93	< 0.001*	<0.001*
1-HEXANOL	GLV	*Nex, Nob, – – –, – – –*	1.00, 0.70, 0.84	< 0.001*	<0.001*
METHYL BENZOATE	Aromatic	*Nex, Nob, – – –, – – –*	0.81, 0.63, 0.72	< 0.001*	<0.001*
BENZYL ALCOHOL	Aromatic	*Nex, Nob, – – –, – – –*	0.92, 0.21, 0.44	0.034*	0.038*
HEXYL BENZOATE	Aromatic	*Nex, Nob, – – –, – – –*	1.00, 0.18, 0.43	0.025*	0.033*
α-COPAENE	Sesquiterpene	*Nex, Nob, – – –, – – –*	1.00, 0.30, 0.55	0.009*	0.011*
α-HUMULENE	Sesquiterpene	*Nex, Nob, Nre, – – –*	0.98, 0.64, 0.79	0.004*	0.005*
1-OCTANOL	GLV	*Nex, Nob, – – –, Nsy*	0.98, 0.83, 0.90	< 0.001*	<0.001*
(*E,E*)-4,8,12-TRIMETHYLTRIDECA-1,3,7,11-TETRAENE	Homoterpene	*Nex, – – –, Nre, – – –*	0.97, 0.61, 0.77	< 0.001*	<0.001*
CARYOPHYLLENE OXIDE	Sesquiterpene	*Nex, – – –, Nre, – – –*	0.91, 0.78, 0.84	< 0.001*	<0.001*
β-CARYOPHYLLENE	Sesquiterpene	*Nex, – – –, Nre, Nsy*	1.00, 0.36, 0.60	0.0377*	0.044*
β-MYRCENE	Monoterpene	*Nex, – – –, – – –, Nsy*	0.99, 0.28, 0.52	0.0064*	0.008*
(*E*)-β-FARNESENE	Sesquiterpene	*Nex, – – –, – – –, Nsy*	0.91, 0.86, 0.89	< 0.001*	<0.001*
(*Z,E*)-α-FARNESENE	Sesquiterpene	*Nex, – – –, – – –, Nsy*	0.90, 0.69, 0.79	< 0.001*	<0.001*
4-METHYL-1-PENTANOL	Aliphatic	*– – –, Nob, – – –, – – –*	1.00, 0.82, 0.91	< 0.001*	<0.001*
UNKNOWN METHYL ESTER 1	Aliphatic	*– – –, Nob, – – –, – – –*	0.10, 1.00, 0.99	< 0.001*	<0.001*
2-PHENYLETHANOL	Aromatic	*– – –, Nob, – – –, – – –*	1.00, 1.00, 1.00	< 0.001*	<0.001*
M/Z 109(100), 41(33), 81(17), 43(16), 104(16), 67(15), 57(14), 79(13), 93(11), 69(11)		*– – –, Nob, – – –, – – –*	0.99, 0.94, 0.97	< 0.001*	<0.001*
METHYL SALICYLATE	Aromatic	*– – –, Nob, Nre, – – –*	0.83, 0.38, 0.56	0.015*	0.018*
HEXADECANE	Hydrocarbon	*– – –, – – –, Nre, – – –*	0.98, 0.65, 0.80	< 0.001*	<0.001*
DOCOSANE	Hydrocarbon	*– – –, – – –, – – –, Nsy*	0.97, 0.90, 0.93	< 0.001*	<0.001*
M/Z 43(100), 41(67), 45(37), 81(28), 55(28), 57(24), 44(21), 93(17), 95(16), 67(16)	Terpene	*– – –, – – –, – – –, Nsy*	1.00, 0.90, 0.95	< 0.001*	<0.001*
GERMACRENE D	Sesquiterpene	*– – –, – – –, – – –, Nsy*	1.00, 0.70, 0.84	< 0.001*	<0.001*
M/Z 43(100), 41(65), 45(48), 57(32), 44(26), 55(22), 81(18), 56(17), 71(16), 69(14)		*– – –, – – –, – – –, Nsy*	1.00, 0.95, 0.98	< 0.001*	<0.001*

*Green leaf volatiles (GLV) are listed under compound class. For each species or group, significant indicator value indices are shown. A is the probability that a specific compound belongs to that species (1 = belongs only to this species) and B is the probability of finding a specific compound in that species (1 = belongs to all samples in this species). Stat is the square root of the product of A and B. Raw P-values and P-values corrected for false discovery rates are provided. Unknown compounds are listed with 10 most abundant MS ion fragments (m/z) in descending order from the base peak (= 100%). Asterisks indicate a significant difference among species (P < 0.05).*

### Leaf Volatile Emissions Are Higher in Induced Plants

**FIGURE 7:**
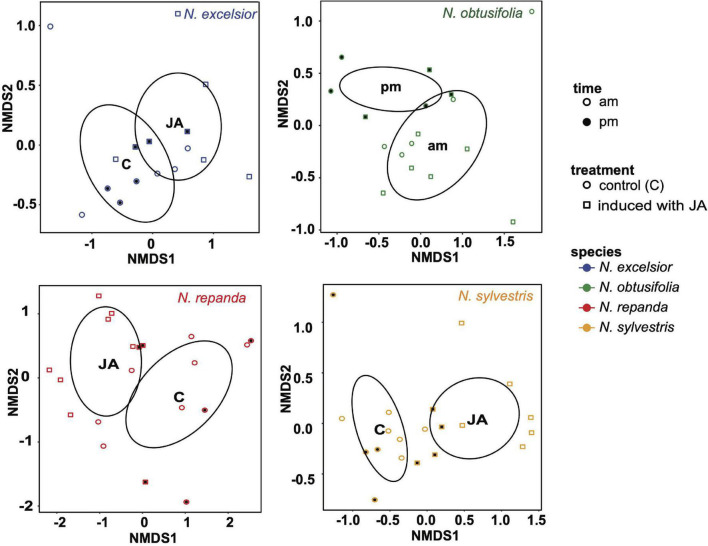
Non-linear multidimensional scaling (NMDS) plot showing clustering associated with induction treatment or time of day differences in leaf volatile chemistry for each species. Ellipses were generated using covariance matrices and displayed for significant groupings. Shape of each point indicates whether leaves were induced with jasmonic acid (square) or control (constitutive; circle). Point fill indicates the time of day volatile emission was measured (morning = unfilled points and evening = filled/black points). *N. excelsior* 2D stress = 0.048, induction treatment *r*^2^ = 0.228, *P* = 0.042; *N. obtusifolia* 2D stress = 0.097, time *r*^2^ = 0.216, *P* = 0.024; *N. repanda* 2D stress = 0.086, induction treatment *r*^2^ = 0.326, *P* = 0.002; *N. sylvestris* 2D stress = 0.081, induction treatment *r*^2^ = 0.494, *P* = 0.001.

Inducible leaf volatile compounds were identified in plants of *N. excelsior, N. repanda*, and *N. sylvestris* that had been JA treated to mimic herbivory prior to volatile collections (Table 2A and Figure 7). These were the same three species that showed significantly inducible anti-herbivore resistance in the larval growth assays on leaf tissue.

**TABLE 2:** Volatile compounds differed in (A) uninduced and induced plants for *Nicotiana excelsior, N. repanda*, and *N. sylvestris* and (B) between morning and night for *N. obtusifolia*.

(A) Inducible volatile compounds.													
Treatment		*N. excelsior*			*N. obtusifolia*			*N. repanda*			*N. sylvestris*		
	Volatile	A, B, stat	*P*	*fdr P*	A, B, stat	*P*	*fdr P*	A, B, stat	*P*	*fdr P*	A, B, stat	*P*	*fdr P*
**Induction With Jasmonic Acid**	m/z 43(100), 67(75), 41(54), 82(46), 71(34), 55(15), 42(13), 57(9), 40(7), 68(6)	0.95, 1.00, 0.97	< 0.001*	0.012*	-	-	-	-	-	-	-	-	-
	(*Z*)-3-HEXENYL BUTYRATE	0.88, 1.00, 0.94	0.002*	0.030*	-	-	-	-	-	-	-	-	-
	UNKNOWN SIMILAR TO METHYL LINOLEATE	0.89, 1.00, 0.94	0.004*	0.047*	-	-	-	-	-	-	-	-	-
	FARNESOL ISOMER 2	0.75, 1.00, 0.87	0.009*	0.092	-	-	-	-	-	-	-	-	-
	(*Z*)-3-HEXENYL ISOVALERATE	0.5, 1.00, 0.86	0.049*	0.271	-	-	-	-	-	-	-	-	-
	UNKNOWN METHYL ESTER 2	0.80, 0.88, 0.83	0.048*	0.271	-	-	-	-	-	-	-	-	-
	(*E*)-β-OCIMENE	0.89, 1.00, 0.94	0.028*	0.218	-	-	-	-	-	-	0.95, 1.00, 0.98	< 0.001*	0.004*
	β-ELEMENE	-	-	-	-	-	-	-	-	-	0.98, 0.80, 0.89	0.001*	0.010*
	β-MYRCENE	-	-	-	-	-	-	-	-	-	0.88, 0.50, 0.66	0.030*	0.102
	(*Z,E*)-α-FARNESENE	-	-	-	-	-	-	-	-	-	0.87, 0.80, 0.83	0.019*	0.080
	Linalool	-	-	-	-	-	-	-	-	-	0.87, 0.70, 0.78	0.020*	0.080
	β-CARYOPHYLLENE	-	-	-	-	-	-	-	-	-	1.00, 0.70, 0.84	0.004*	0.021*
	(*E,E*)-α-FARNESENE	-	-	-	-	-	-	0.91, 0.90, 0.91	0.003*	0.025*	0.99, 1.00, 0.99	< 0.001*	0.003*
	CARYOPHYLLENE OXIDE	-	-	-	-	-	-	0.92, 1.00, 0.96	< 0.001*	0.005*	0.95, 0.80, 0.87	0.002*	0.016*
	(*E*)-α-BERGAMOTENE	-	-	-	-	-	-	1.00, 0.60, 0.78	0.010*	0.050	1.00, 0.50, 0.71	0.033*	0.102
	(*Z*)-JASMONE	-	-	-	-	-	-	0.97, 0.60, 0.76	0.011*	0.050	-	-	-
	(*E,E*)-4,8,12-TRIMETHYLTRIDECA-1,3,7,11-TETRAENE (TMTT)	-	-	-	-	-	-	0.86, 0.90, 0.88	0.006*	0.041*	-	-	-
	α-HUMULENE	-	-	-	-	-	-	0.95, 1.00, 0.98	< 0.001*	0.005*	-	-	-
	(*Z*)-3-HEXENYL-BENZOATE	-	-	-	-	-	-	0.73, 0.90, 0.81	0.047*	0.159	-	-	-
**(B) Day And Night Volatile Compounds.**													
**Treatment**		***N. excelsior***			***N. obtusifolia***			***N. repanda***			***N. sylvestris***		
	**Volatile**	**A, B, stat**	***P***	***fdr P***	**A, B, stat**	***P***	***fdr P***	**A, B, stat**	***P***	***fdr P***	**A, B, stat**	***P***	***fdr P***

AM	2-PHENYLETHANOL	-	-	-	0.65, 1.00, 0.80	0.013*	0.193	-	-	-	-	-	-
AM + DARK AM	(*Z*)-3-HEXEN-1-OL	-	-	-	0.96, 0.82, 0.89	0.009*	0.092	-	-	-	-	-	-
	α-COPAENE	-	-	-	1.00, 0.64, 0.80	0.033*	0.188	-	-	-	-	-	-
	FARNESOL ISOMER 2	-	-	-	1.00, 0.64, 0.80	0.036*	0.188	-	-	-	-	-	-
DARK AM	HEXYL BENZOATE	-	-	-	0.90, 0.75, 0.82	0.009*	0.092	-	-	-	-	-	-
PM	α-HUMULENE	-	-	-	0.98, 0.67, 0.81	0.026*	0.193	-	-	-	-	-	-

*For volatiles associated with morning, compounds are broken down based on the lighting conditions (normal lit conditions and/or in dark conditions during AM). The significant indicator value indices are shown in columns for each species: A is the probability that a specific compound belongs to the group (1 = belongs only to this group) and B is the probability of finding a specific compound in the group (1 = belongs to all samples in this group). Stat is the square root of the product of A and B. Uncorrected P-values and P-values corrected for false discovery rates are provided. Unknown compounds are listed with 10 most abundant MS ion fragments (m/z) in descending order from the base peak (= 100%). Asterisks indicate a significant difference between treatments within a species (P < 0.05).*

Volatiles of *N. obtusifolia* were similar among induced and uninduced plants but varied slightly between day and night (Table 2B and Figure 7). Volatile collections done in the dark during the daytime (to mimic the plant conditions during moth foraging assays) showed volatile chemistry similar to normal illuminated daytime conditions. Only one compound (hexyl benzoate) was associated with dark day conditions in *N. obtusifolia* (Table 2). Overall emission rates (ng scent/h) tended to be higher in the morning volatile collections than the night collections (ANOVA: time of day *F*_1,65_ = 7.38, *P* = 0.008, species *F*_3,65_ = 14.7, *P* < 0.001, time*species *F*_3,65_ = 2.07, *P* = 0.11) but this difference was only significant in *N. repanda* (Tukey *post hoc* test am-pm: *N. repanda P*_adjusted_ = 0.03) (Supplementary Table 2).

The chemical profile of induced *N. excelsior* plants was dominated by higher production of a lipoxygenase pathway “green leaf volatile” (GLV), (*Z*)-3-hexenyl butyrate, and an unknown compound (KI 2604). Three volatile compounds were associated with induction in both *Nicotiana repanda* and *N. sylvestris*. For *N. repanda* and *N. sylvestris*, two compounds were only identified in induced plants and were not present in quantifiable amounts in uninduced plants [(*E*)-α-bergamotene in both species and β-caryophyllene in *N. sylvestris*] (Table 2). Overall emission rates (ng scent/h) differed among species and with JA induction treatment (ANOVA: induction *F*_1,65_ = 36.3, *P* < 0.001, species *F*_3,65_ = 20.6, *P* < 0.001, induction*species *F*_3,65_ = 4.91, *P* = 0.004). *Nicotiana excelsior* had the highest overall emission rate (Tukey *post hoc* test: *P*_adjusted_ < 0.001). Induced plants of *Nicotiana repanda* and *N. sylvestris* had higher overall emission rates than conspecific control plants (Tukey *post hoc* test: *N. repanda P*_adjusted_ < 0.001, *N. sylvestris P*_adjusted_ = 0.02) (Figure 6 and Supplementary Table 2).

## Discussion

In this study, we show that the pollinating-herbivore *Manduca sexta* uses leaf volatiles and cues of anti-herbivore resistance to inform adult feeding and oviposition behaviors on host plants. Floral traits play a clear role in moth foraging and oviposition choices because of the direct interactions between flowers and pollinators (Adler and Bronstein, 2004). We show that vegetative cues of plant resistance also influence adult moth behavior and preference in ways that affect offspring success. Adult oviposition choices were consistent with measurements of larval performance, suggesting that females respond to selective pressure to avoid well defended host plants. In contrast, foraging decisions did not align with oviposition preference and larval performance. When *M. sexta* adult females exhibited a foraging preference, they preferred to forage from the artificial flowers on the induced vegetative background compared with the uninduced (constitutive) background, which represented increased overall emissions. These results provide important evidence that plant vegetative induction alters leaf anti-herbivore resistance and volatiles in ways that affect pollinator behavior even in the absence of variation in floral cues.

### Tobacco Species Present Varying Challenges to *Manduca sexta* Larvae and Ovipositing Adults

One benefit of studying tobacco-hornworm interactions as a model system is the wealth of prior studies available to provide inference for comparative studies such as ours. For example, a decade of careful lab studies of *N. sylvestris* demonstrated that leaf damage induces systemic increases in leaf nicotine (Baldwin, 1988), that these changes are mediated by jasmonates (JA and its methyl ester, MeJA; Baldwin et al., 1997; Ohnmeiss et al., 1997) and that *M. sexta* larvae move away from such plants (despite their relative resistance to nicotine; van Dam et al., 2000). These studies culminated with a clear demonstration that leaf nicotine contributes significantly to herbivore resistance when *M. sexta* larvae showed increased leaf consumption and growth rates on plants producing low levels of nicotine (Voelckel et al., 2001). In the context of these studies, the induced resistance shown by *N. sylvestris* in our study (Figure 2) may have been greater due to our cross-experimental standardized JA treatment instead of actual larval herbivory. McCloud and Baldwin (1997) showed that larval *M. sexta* salivary regurgitant, when applied to mechanically wounded leaves, decouples (high) leaf JA response from root nicotine induction in *N. sylvestris*, which they interpreted as a co-evolutionary response of this host-specialized moth species. In our study, *N. repanda* plants showed the highest induced resistance to *M. sexta* larvae, consistent with earlier studies demonstrating that wounding + MeJA induced rapid deposition and acylation of nornicotine in leaf trichomes, where it is 1000-fold more toxic than nicotine to *M. sexta* larvae (Laue et al., 2000). This acylation may explain why *N. repanda* plants showed stronger induced resistance to *M. sexta* larvae than did *N. obtusifolia* plants (Figure 2), given that leaf damage has been shown to induce nornicotine accumulation in both species (Baldwin and Ohnmeiss, 1993). Thus, the measured resistance levels in the three American species selected for our study confirm the varying responses to herbivory by *M. sexta* larvae seen in previous studies.

As was true for traits related to direct resistance, many of the VOCs measured in this study, from the series of (*Z*)-3-hexenyl-related GLVs to β-caryophyllene, α-humulene and farnesene isomers, were previously identified from the vegetative headspace of related tobacco species, including *N. tabacum* (Andersen et al., 1988; De Moraes et al., 2001). While these compounds are common herbivore induced VOCs associated with indirect defense in other plant families (Paré and Tumlinson, 1999), the diterpene neophytadiene, which was a dominant VOC in many of our samples, is a characteristic fragrance note of tobacco leaves (Uegaki et al., 1980) but is seldom encountered outside the Solanaceae (Knudsen et al., 2006). Again, our findings may have been conservative due to our decision to use JA instead of *M. sexta* larval regurgitant to induce plant responses in our experiments. Recent studies have shown that the oral secretions of larval *M. sexta* activate leaf enzymes that convert (*Z*)-3-hexenyl GLVs to (*E*)-2-hexenyl volatiles, and that this conversion results in the attraction of natural enemies to consume larvae on *Nicotiana attenuata* (Allmann and Baldwin, 2010) while reducing *M. sexta* oviposition on *Datura wrightii* (Allmann et al., 2013). Our use of JA to induce our experimental plants is the likely explanation for the dominance of (*Z*)-3-hexenyl compounds and absence of (*E*)-2-hexenyl compounds in our trapped headspace blends (Supplementary Table 1).

### Plant Vegetative Volatiles Affect Insect Preference and Performance

Although there was overlap in the constitutive and induced volatiles produced by the four *Nicotiana* species, we show that the species differed in their overall levels of resistance and their volatile composition in ways that affected insect preference and performance. Previous studies have shown that nectar foraging behavior by flower-naïve *M. sexta* moths is gated by plant volatiles (Raguso and Willis, 2002) and that some compounds or blends thereof are more attractive than others (Riffell et al., 2009; Bisch-Knaden et al., 2018). Nevertheless, this requirement is not strictly constrained, given that *M. sexta* show feeding responses to artificial flowers coupled with vegetative odorants from *Nicotiana* and *Datura* host plants (Cutler et al., 1995; Raguso and Willis, 2005; Raguso et al., 2007).

Volatiles can be particularly important in determining insect feeding and oviposition decisions because they allow an insect to assess cues of plant damage or defense before coming into physical contact with the plant. Herbivory can result in changes in both leaf and floral volatiles that insects can use to assess plant resistance and rewards (Lucas-Barbosa et al., 2015). Therefore, if volatile cues are reliable indicators of plant defenses that negatively affect pollinator fitness, pollinators are likely to discriminate against plants with higher levels of constitutive or induced volatiles. Of the four species examined in this study, *Nicotiana repanda* demonstrated the highest constitutive anti-herbivore resistance in the larval growth rate assays while *N. obtusifolia, N. excelsior*, and *N. sylvestris* all had lower levels of resistance. The variation in levels of induced anti-herbivore resistance (Figure 1) and induced vegetative volatiles across species (Table 1) combined with the differences in evolutionary history between the *Nicotiana* species and their native pollinators and herbivores likely influenced the ways that vegetative induction shaped the observed foraging and oviposition patterns.

### Oviposition Preference for Uninduced Plants Aligns With Optimal Oviposition Theory

For all four *Nicotiana* species tested, *M. sexta* oviposition preference aligned with higher larval performance on control (uninduced) leaves compared with induced leaves, indicating that the jasmonic acid induction treatment not only evoked an induced plant response but that this response was sufficient to inform adult moth behavior and alter larval performance even without floral cues reflecting plant resistance. More eggs were laid on the uninduced versus induced plants when adult *M. sexta* females were offered a choice between the two plants for oviposition (Figure 4). This was the case for the three inducible species (*N. excelsior, N. repanda*, and *N. sylvestris*) as well as for *N. obtusifolia*, despite no significant differences in larval growth (Figure 2) or volatile emissions between control and induced *N. obtusifolia* plants (Figure 7 and Table 2). This finding is consistent with the predictions of optimal oviposition theory (Thompson, 1988). Because of the clear relationship between leaf defensive status and larval performance, this preference for oviposition on control leaves could improve offspring performance in the absence of other factors like predator avoidance that could favor feeding on defended leaves (Jaenike, 1978; Thompson, 1988; Gripenberg et al., 2010).

### Induced Vegetative Volatiles Did Not Deter Pollinator Foraging

From the plant perspective, herbivore deterrence *via* defended leaves is likely to be beneficial for plant fitness as long as this defense does not deter pollinators (Irwin, 2010). In contrast to the observed relationships between larval performance, adult oviposition choice, and leaf induction status, there was no correlation between leaf induction and adult foraging preference for three of the four species, *N. obtusifolia, N. repanda*, and *N. sylvestris*. In *N. excelsior*, adult females actually foraged more frequently from the artificial flower on the induced vegetative background. Although floral volatiles and nectar taste cues (absent in this study) are known to play a role in foraging preferences (Adler and Irwin, 2005), this result indicates that vegetative volatiles alone can impact foraging preferences and can do so in ways that do not align with oviposition preferences and larval performance.

### Volatile Differences May Reflect the Plant-Insect Coevolutionary Histories of the Species

The differences in moth foraging preferences on the *Nicotiana* species are likely the result of the variation in volatile profiles as well as different plant-insect coevolutionary histories. *Nicotiana obtusifolia, N. repanda*, and *N. sylvestris* are found across the Americas where *Manduca sexta* is a voracious herbivore and/or pollinator of Solanaceous species and is known to have important effects on host plant fitness (McCall et al., 2020). In contrast, *N. exclesior* belongs to the monophyletic Suaveolentes section in Australia and is undergoing rapid radiation (Knapp et al., 2004; Dodsworth et al., 2020). In its native range, *N. excelsior* co-occurs with pollinating hawkmoths other than *M. sexta* (Moulds et al., 2020), and the absence of selective pressure from *M. sexta* may have contributed to the differences in resistance and volatiles among the species. Moreover, *Nicotiana excelsior* has a more diverse induced leaf volatile profile than the other three species studied here (Tables 1, 2) and the increase in overall volatile complexity or emission rates could have made it easier for moths to locate the induced plant. Some of the compounds we identified in *N. excelsior* leaves have also been identified in the leaves and floral tissues of other tobacco species, including the related Australian species, *N. suaveolens* (Effmert et al., 2008), and the presence of floral-type volatiles in the leaves could be attractive for foraging insects. For example, the emission of (*E*)-α-bergamotene from *Nicotiana attenuata* flowers promotes flower probing and pollination effectiveness by *M. sexta*, and leaf emissions of the same compound attract predators to eggs oviposited by female moths (Zhou et al., 2017). In our study, we identified (*E*)-β-ocimene as one of the inducible compounds present in *N. excelsior* (Table 2) and isomers of this compound are found in both floral and vegetative tissues and are known to play a role in both pollinator attraction and anti-herbivore defense (Farré-Armengol et al., 2017). These results underscore the complex roles that plant resistance and volatiles play in mediating interactions between plants and their insect herbivores and pollinators, particularly when the same insect species serves both mutualist and antagonistic roles (Ramos and Schiestl, 2019).

### Plant Induction Status Affected Vegetative Volatiles to a Greater Extent Than Diel Variation

Although temporal and diel variation in emission rate/timing of leaf and floral volatiles is predicted to help minimize the plant costs of deterring herbivores while maximizing pollinator attraction (Euler and Baldwin, 1996; De Moraes et al., 2001; Reisenman et al., 2013), we only observed significant variation in day and night vegetative volatiles in one of the four species, *N. obtusifolia*. It is possible that different conditions (such as varying day and night temperatures or signals of damage during different times of day) would result in stronger diel rhythms of volatile production like those seen in other *Nicotiana* species (Raguso et al., 2003). De Moraes et al. (2001) found that *Nicotiana tabacum* showed strong differences in herbivore induced volatiles during night and day and that different leaf herbivores induced similar volatile components. However, in our study the small differences in volatile chemistry in *N. obtusifolia* during different lighting and time of day settings are unlikely to have influenced moth behavior during the foraging and oviposition assays. In the case of *N. obtusifolia*, slight volatile variation or additional touch/taste/visual cues associated with oviposition choice likely led to avoidance of induced plants for egg laying (Baur et al., 2003; Reisenman et al., 2009).

### Correlations Between Leaf and Floral Volatiles Can Influence Insect Behavior

Insect foraging and oviposition choice can directly and indirectly influence offspring performance and fitness. In pollinating-herbivore species, adult fitness is affected by the presence of deterrent secondary compounds in nectar and larval fitness is affected by the defensive compounds in the tissues on which neonates hatch and feed. Floral volatiles may contain both attractive and repellant chemicals depending on the types of insects interacting with the plant, including pollinators, folivores, and/or florivores (Farré-Armengol et al., 2015; Rusman et al., 2019; Haas and Lortie, 2020). Whether adult pollinators use leaf and/or floral volatiles to determine foraging and oviposition choice is likely influenced by correlations between leaf and floral defense. Across *Nicotiana*, leaf and floral levels of defensive compounds such as nicotine and anabasine, are not correlated in all species (Adler et al., 2012). In *Nicotiana suaveolens*, an Australian species, leaf induction of secondary chemicals *via Manduca* larval herbivory does not alter floral volatile composition or emission rates (Effmert et al., 2008). When floral and leaf secondary compounds are not correlated, an insect’s ability to assess both vegetative and floral cues is likely to be especially important for maximizing fitness *via* diet (foraging) and offspring success (oviposition choice). Previous studies simultaneously presenting *M. sexta* with leaf and floral volatiles from different plant species have indicated that floral and vegetive odor can act synergistically to affect moth behavior (Kárpáti et al., 2013). Thus, insect behavior is the result of complex integration of vegetative and floral cues indicating host plant suitability, which may be context-dependent based on abiotic and biotic conditions. Future work manipulating both vegetative and floral volatiles should aim to determine the relative contributions of vegetative and floral volatiles on pollinator behavior and resulting plant fitness.

## Data Availability Statement

The raw data supporting the conclusions of this article will be made available by the authors, without undue reservation.

## Author Contributions

DJ designed the study and carried out moth growth and behavioral trials, performed statistical analyses for the growth and behavioral experiments, and designed the figures. DJ and RR collected plant volatiles in the greenhouse and performed statistical analyses on the GC volatile data and contributed to the writing of the manuscript. RR processed the volatile samples to generate the GC-MS and GC-FID data. All authors contributed to the article and approved the submitted version.

## Conflict of Interest

The authors declare that the research was conducted in the absence of any commercial or financial relationships that could be construed as a potential conflict of interest.

## Publisher’s Note

All claims expressed in this article are solely those of the authors and do not necessarily represent those of their affiliated organizations, or those of the publisher, the editors and the reviewers. Any product that may be evaluated in this article, or claim that may be made by its manufacturer, is not guaranteed or endorsed by the publisher.

## Footnotes

1
https://webbook.nist.gov/

## Supplementary Material

Supplementary Table 1 Volatile compounds and class, retention time (RT) for the GC-MS and GC-FID, Kovat’s index (KI), evidence for volatile identification*, and normalized peak area ± standard error for plant anti-herbivore resistance treatment [control (C) or induced with jasmonic acid (JA)], and time of day (day or night) broken down by *Nicotiana* species. GC-FID peak areas were normalized by dividing by the peak area of the tridecane internal standard. Bold values indicate significant differences between control/induced or day/night emissions from the indicator value indices and asterisks indicate significant differences after corrections for false discovery rate (*P* < 0.05).

Supplementary Table 2 Mean volatile emission rates, standard errors (se), and sample size (N) for the four *Nicotiana* species broken down by plant anti-herbivore resistance treatment [control (C) or induced with jasmonic acid (JA)], and time of day (day or night). The total volatile emission rate (ng scent / h) for each plant was calculated as: [(sum of all normalized GC-FID peak areas) × amount of internal standard injected (34 ng) × sample volume (55 μL)] / timespan of volatile collection (5 h).
